# Antimicrobial peptide thanatin fused endolysin PA90 (Tha-PA90) for the control of *Acinetobacter baumannii* infection in mouse model

**DOI:** 10.1186/s12929-024-01027-4

**Published:** 2024-04-15

**Authors:** Jeonghyun Lim, Heejoon Myung, Daejin Lim, Miryoung Song

**Affiliations:** 1 Department of Bioscience and Biotechnology, Hankuk University of Foreign Studies, Yongin, 17035 Republic of Korea; 2 LyseNTech Co., Ltd., Seongnam-Si, 13486 Republic of Korea; 3 Division of Biomedical Convergence, College of Biomedical Science, Kangwon National University, Chuncheon, 24341 Republic of Korea

**Keywords:** Endolysin, Thanatin, Tha-PA90, Designed endolysin, *A*. *baumannii* infection, Drug-resistant gram-negative pathogen

## Abstract

**Background:**

This study addresses the urgent need for infection control agents driven by the rise of drug-resistant pathogens such as *Acinetobacter baumannii*. Our primary aim was to develop and assess a novel endolysin, Tha-PA90, designed to combat these challenges.

**Methods:**

Tha-PA90 incorporates an antimicrobial peptide (AMP) called thanatin at its N-terminus, enhancing bacterial outer membrane permeability and reducing host immune responses. PA90 was selected as the endolysin component. The antibacterial activity of the purified Tha-PA90 was evaluated using an in vitro colony-forming unit (CFU) reduction assay and a membrane permeability test. A549 cells were utilized to measure the penetration into the cytosol and the cytotoxicity of Tha-PA90. Finally, infection control was monitored in *A. baumannii* infected mice following the intraperitoneal administration of Tha-PA90.

**Results:**

Tha-PA90 demonstrated remarkable in vitro efficacy, completely eradicating *A. baumannii* strains, even drug-resistant variants, at a low concentration of 0.5 μM. Notably, it outperformed thanatin, achieving only a < 3-log reduction at 4 μM. Tha-PA90 exhibited 2–3 times higher membrane permeability than a PA90 and thanatin mixture or PA90 alone. Tha-PA90 was found within A549 cells' cytosol with no discernible cytotoxic effects. Furthermore, Tha-PA90 administration extended the lifespan of *A. baumannii*-infected mice, reducing bacterial loads in major organs by up to 3 logs. Additionally, it decreased proinflammatory cytokine levels (TNF-α and IL-6), reducing the risk of sepsis from rapid bacterial lysis. Our findings indicate that Tha-PA90 is a promising solution for combating drug-resistant *A. baumannii*. Its enhanced efficacy, low cytotoxicity, and reduction of proinflammatory responses render it a potential candidate for infection control.

**Conclusions:**

This study underscores the significance of engineered endolysins in addressing the pressing challenge of drug-resistant pathogens and offers insights into improved infection management strategies.

**Supplementary Information:**

The online version contains supplementary material available at 10.1186/s12929-024-01027-4.

## Background

In recent years, infection control strategies have been significantly impeded by the escalating rates of antibiotic resistance. Multi-drug-resistant (MDR) and extensively drug-resistant (XDR) bacteria have rapidly emerged, with reports indicating the existence of pandrug-resistant (PDR) bacteria [1]. Among these, *Acinetobacter baumannii* has emerged as one of the most dangerous pathogens, responsible for a spectrum of nosocomial infections including wound infections, urinary tract infections, bacteremia, secondary meningitis, and ventilator-associated pneumonia [2]. *A. baumannii* is now categorized by the World Health Organization (WHO) as a “high priority” pathogen owing to its diverse resistance mechanisms and the rapid emergence of MDR, XDR, and PDR strains [3, 4]. These resistance mechanisms include the production of antimicrobial hydrolyzing enzymes, altering outer membrane proteins to restrict access to targets, and mutations affecting target sites, including efflux pump production [2, 5].

Bacteriophages have garnered renewed attention in response to the escalating antibiotic resistance crisis as an alternative approach for combating MDR bacterial infections. These viruses can infect and eliminate their specific host bacteria once progeny phages are produced [6]. Despite the inherent advantages of phages, such as high specificity, lack of cytotoxicity against eukaryotic cells, and minimal impact on the microbiome [7], phage therapy is limited by challenges associated with a narrow host spectrum, the emergence of phage resistance and challenges related to clinical preparation [8, 9]. Endolysins have shown promise in addressing these challenges. In nature, endolysins are produced by bacteriophages after their replication cycle to hydrolyze peptidoglycan, a bacterial cell wall component, facilitating the release of progeny phages [10, 11]. When applied extracellularly, purified endolysins have demonstrated potent antibacterial and anti-biofilm activity against gram-positive pathogens [12, 13]. Notably, endolysins possess attributes such as non-toxicity to mammalian cells, a broad target spectrum, and a reduced likelihood of encountering resistance mechanisms [14]. However, the principal hurdle in developing endolysins against gram-negative pathogens lies in an outer membrane that hinders endolysins’ access to their substrate, peptidoglycan [7, 15]. Recent research efforts have focused on engineering native endolysins to ensure antimicrobial activity against gram-negative bacteria, including MDR strains. These innovations have circumvented the outer membrane barrier by incorporating outer membrane-disrupting or polycationic peptides [16, 17].

Antimicrobial peptides (AMPs) are generally short, cationic peptides produced by various organisms, including insects, humans, and plants. Most AMPs exhibit antimicrobial and immune-modulating activities with limited susceptibility to resistance [18, 19]. Owing to their positive charge, AMPs interact with negatively charged membrane components, such as lipopolysaccharides (LPSs), leading to membrane disruption and bacterial cell death [19–21]. Thanatin, a 21-amino-acid cationic AMP derived from insects, features a disulfide bond between Cys11 and Cys18 [22]. It replaces divalent cations bound to LPSs, thereby disrupting the outer membrane of gram-negative bacteria [23, 24]. Subsequently, thanatin gains access to the periplasmic space and interacts with the Lpt protein complex, thereby inhibiting LPS translocation [25]. Furthermore, it exhibits potent antibacterial activity against gram-negative pathogens in a mouse sepsis model and neutralizes released LPS [22].

In this study, a designed endolysin was rigorously evaluated in vitro and in vivo to develop a control agent for *A. baumannii* infections. To this end, a native endolysin PA90 was selected as only minor antibacterial activity against gram-negative bacteria, including *Pseudomonas,* was observed; however, PA90 was identified from *Pseudomonas* phage as shown in our previous study [26]. Thanatin peptide was strategically introduced at the N-terminus of PA90 to mitigate the potential adverse effects of LPS release upon bacterial lysis by the endolysin in vivo. Purified thanatin-fused PA90 (Tha-PA90) demonstrated potent bactericidal activity against *A. baumannii* strains, including MDR variants, in vitro. Notably, Tha-PA90 exhibited superior antibacterial activity and cell permeability compared to thanatin alone while also displaying no cytotoxicity to A549 cells. In a mouse model of *A. baumannii* systemic infection, introducing Tha-PA90 improved mouse survival rates and reduced bacterial loads in various organs. Furthermore, administration of Tha-PA90 decreased the expression and serum secretion of proinflammatory cytokines in infected mice. This study offers valuable insights for developing endolysin-based antimicrobials that target gram-negative pathogens, irrespective of their resistance profiles.

## Methods

### Bacterial strains, growth conditions and reagents

All tested bacterial strains were procured from the American Type Culture Collection (ATCC) and the Korean Collection for Type Cultures (KCTC). Clinical isolates of *A. baumannii* were acquired from the Kyungpook National University Hospital National Culture Collection for Pathogens (KNUH-NCCP). DH5α was employed for the cloning process, while SoluBL21™ was utilized for protein purification. Cultures of all bacterial strains were cultivated in Luria Bertani (LB; MBcell #MB-L4488) broth at 37 °C, with agitation at 200 rpm. Ampicillin was supplemented at 100 μg/ml concentration when deemed necessary. Recombinant protein expression was induced by adding isopropyl-B-D-thiogalactopyranoside (IPTG; Biosesang #I1006) at the specified concentrations.

### Purification of recombinant endolysin PA90 or Tha-PA90

The plasmid encoding thanatin-fused PA90 (Tha-PA90) was generated through polymerase chain reaction (PCR), utilizing the pAS033 plasmid as a template. The pAS033 plasmid was designed to overexpress cell-penetrating peptide DS4.3-fused pA90 on the pET21a backbone [26]. Notably, the DNA sequence encoding the Thanatin peptide (GSKKPVPIIYCNRRTGKCQRM) was incorporated into the primer sequences (Additional file 1: Table S1, underlined). Following sequencing analysis to confirm the construct, the resultant plasmid, pAS036, was introduced into SoluBL21™ for the subsequent purification of Tha-PA90.

Cultures of SoluBL21™ carrying either pET21a::PA90 (pAS025) [26] or pAS036 were cultivated in 1.5 L of LB broth supplemented with ampicillin (100 μg/ml) at 37 °C with vigorous aeration at 200 rpm. Once the optical density at 600 nm reached 0.6, IPTG was introduced into the culture to induce protein expression. Following a 5-h incubation at 37 °C in a shaking incubator, the cells were harvested by centrifugation at 5000 × g for 10 min at 4 °C. Subsequently, cell lysis was achieved via sonication, utilizing the resuspended pellet in 100 ml of lysis buffer (comprising 20 mM Tris–HCl, pH 8.5, 0.5 M NaCl, and 10 mM imidazole). After eliminating unbroken cells through centrifugation at 14,000 × g for 30 min at 4 °C, the supernatant fraction was filtered through a 0.4 μm-pore size filter (GVS #FJ25ASCCA004FL01). The subsequent step involved applying each collected fraction to a 5 ml HisTrap HP column (Cytiva #17,524,802), installed in an ÄKTA fast protein liquid chromatography (FPLC) system (Cytiva, USA), and controlled using UNICORN 5.1 software. The resin-bound endolysins were eluted using a gradient of imidazole concentration (10 mM–0.5 M) in a buffer of 20 mM Tris–HCl, pH 8.5, and 0.5 M NaCl. Subsequently, the eluted fraction was diluted in 20 mM Tris–HCl, pH 8.5 buffer for application to a 5 ml HiTrap SP column (Cytiva #17–1152-01) for further purification. Elution from the HiTrap SP column was conducted using 20 mM Tris–HCl, pH 8.5 buffer containing 0.6 M NaCl. Finally, the fractions obtained were dialyzed overnight at 4 °C in a 20 mM Tris–HCl, pH 7.5, and 150 mM NaCl solution, utilizing Snakeskin™ dialysis tubing (Thermoscientific #68,700). Filtration through a 0.4 μm-pore size filter (GVS #FJ25ASCCA004FL01) was performed to remove aggregated proteins. The quantification of each endolysin was determined using the Bradford assay kit (Biorad #5,000,006), with bovine serum albumin (BSA; Promega, #R396A) serving as the standard.

### Determination of the lytic spectrum of Tha-PA90

The antibacterial activity of Tha-PA90 was assessed through a CFU reduction assay, employing *A. baumannii*, *Escherichia coli*, *Pseudomonas aeruginosa*, *Salmonella Typhimurium*, *Enterobacter cloacae*, and *Klebsiella pneumoniae* as the target strains. Bacterial cultures were allowed to reach the mid-exponential growth phase of OD_600_ = 0.8 to initiate the assay. Subsequently, the cells were harvested by centrifugation at 3500 × g for 5 min. The harvested cells were adjusted to 1 × 10^6^ CFU in a 20 mM HEPES solution at pH 7.4 and mixed with varying concentrations of Tha-PA90 (0, 0.0625, 0.125, 0.25, and 0.5 μM) in a 96-well plate. Following a 2-h incubation at 37 °C, the cells were diluted in 1 × phosphate-buffered saline (PBS) and placed onto LB agar plates to facilitate the enumeration of surviving bacteria.

### Permeability test by 1-N-phenylnaphthylamine uptake assay

Bacterial membrane permeability was assessed via a 1-N-phenylnaphthylamine (NPN) uptake assay [27]. *A. baumannii* ATCC 19606 strain was cultured in LB until the optical density at 600 nm reached 0.4. The harvested cells were washed and resuspended in a 5 mM HEPES solution at pH 7.2. The CFU of the cells were adjusted to approximately 1 × 10^8^ CFU/ml. These cells were added to the wells of a 96-well black plate containing a mixture of 50 μL of 40 μM NPN (Sigma-Aldrich #104,043) and 50 μL of 2 μM Tha-PA90. PA90, thanatin peptide (AnyGen, South Korea), or a combination of thanatin peptide and Tha-PA90 was also tested for comparative purposes. As a positive control, 1 mM EDTA was used, while the buffer containing only NPN was the negative control. The plate was incubated at 37 °C for 30 min. Fluorescence was measured at an excitation wavelength of 350 nm and an emission wavelength of 420 nm using a microplate reader (TECAN Infinite M PLEX). The NPN uptake factor was calculated by dividing the fluorescence value of the cell suspension with the buffer containing only NPN after subtracting the fluorescence value of the cell suspension.

### Detection of internalized Tha-PA90 in mammalian cells by western blot analysis

Adenocarcinomic human alveolar basal epithelial cells A549 were cultured in Dulbecco’s modified Eagle’s medium (DMEM; HyClone #SH30243.01) supplemented with 10% fetal bovine serum (FBS; Welgene #S001-01, South Korea) and 1% penicillin–streptomycin (Gibco #15,140,122) at 37 °C in a 5% CO_2_ incubator. A549 cells were seeded at 1 × 10^5^ cells/well density and treated with 1, 5, or 10 μM of Tha-PA90 at 37 °C for 30 min. The same concentrations of PA90 were also applied for comparison. After washing with 1 × Dulbecco’s phosphate-buffered saline (DPBS) 5 times to eliminate external proteins, whole cell lysates were obtained by lysing the cells using 0.05% triton X-100 (v/v of 1 × DPBS). Centrifugation at 13,000 × g for 20 min was performed to remove cell debris, and the supernatant was filtered through a 0.4 μM-pore-size syringe filter (GVS #FJ25ASCCA004FL01). The filtered supernatant was subjected to 10% SDS-PAGE, and the gel was subsequently transferred to a PVDF membrane (GE Healthcare #10,600,022). The membrane was blocked with 5% skim milk (w/v in TBST) at room temperature (RT) for 30 min. Internalized endolysins were detected using an anti-his antibody (Thermofisher #MA1-21,315-1MG) as the primary antibody. Beta-actin was detected using an anti-beta-actin antibody (Cell Signaling #4967S). After incubation at RT for 1 h, the membranes were washed three times with 1 × TBST and incubated for 1 h with HRP-conjugated secondary antibodies: anti-mouse IgG for anti-his (Sigma-Aldrich #12–349) and anti-rabbit IgG for anti-beta-actin (Cell signaling #7074S). Following another round of washing with 1 × TBST, signals were detected using ECL Western Blotting Substrate (Thermo Scientific #32,209) on an iBright imaging system (Thermo Scientific).

### Evaluation of cytotoxicity of Tha-PA90 using LDH assay

The effect of Tha-PA90 on A549 cells was assessed utilizing the lactate dehydrogenase (LDH) PLUS Cytotoxicity Assay kit (DYNEBIO #GBL-P1000). A549 cells were cultured in a 24-well plate, with an initial seeding density of 1 × 10^5^ cells per well, and maintained at 37 °C in a 5% CO_2_ humidified incubator. Following washing with 1 × DPBS, the cells were exposed to varying concentrations of Tha-PA90 (0, 2.5, 5, and 10 μM) for 24 h in DMEM. Mammalian cell lysis buffer served as the positive control. After incubation, the supernatant was transferred into a new 96-well plate and combined with 100 μL of the LDH test reagent. This mixture was subsequently incubated for 30 min, and the addition of stop buffer stopped the reaction. Absorbance was measured at 490 nm using a microplate reader (TECAN Infinite M PLEX). Cytotoxicity was expressed as a percentage, with untreated cells serving as the negative control for the calculation.

### In vivo efficacy of Tha-PA90 in mouse *A. baumannii* infection model

All mouse experiments were conducted according to the guidelines established by the Institutional Mouse Use and Care Committee of Hankuk University of Foreign Studies (HUFS-2021–0003). Seven-week-old female BALB/c mice were procured from Raonbio (South Korea). Cyclophosphamide monohydrate (CPM; 3 mg/mouse; Glentham, #GK2037-1G) was administered via the intraperitoneal route 4 days and 1 day before bacterial infection. *A. baumannii* ATCC 19606 was cultured in brain heart infusion (BHI) media at 37 °C for 18 h with agitation at 200 rpm. Bacterial cells were harvested by centrifugation at 3,500 × g for 5 min and resuspended in 1 × PBS. The CPM-pretreated mice were injected intraperitoneally with 1 × 10^8^ CFU of *A. baumannii*. PA90 or Tha-PA90 at a dosage of 600 μg per mouse was intraperitoneally administered 1 h post infection. In the control experiment (mock), mice were injected with 1 × PBS. Throughout the experiment, the health and survival of the mice were monitored.

The mice were euthanized under anesthesia using a combination of ketamine (200 mg/kg) and xylazine (10 mg/kg) at 10 h dpi. Each organ, including the heart, spleen, liver, kidney, and lung, was aseptically isolated and homogenized in 1 × PBS containing 0.05% Triton X-100, utilizing a T10 ULTRA-TURRAX homogenizer (IKA). Homogenate samples were plated on LB plates containing trimethoprim (5 μg/ml; Sigma #T7883). After overnight incubation at 37 °C, the bacterial numbers were enumerated and expressed as CFU/g of organ or CFU/ml of blood.

### Measurement of cytokine levels in *A. baumannii*-infected mouse upon Tha-PA90 treatment

Serum was obtained by subjecting blood samples from *A. baumannii*-infected mice, treated with Tha-PA90 as previously described, to centrifugation for 30 min at 3000 × g at 4 °C. The levels of IL-6 and TNF-alpha in the serum were quantified using the Mouse IL-6 Uncoated ELISA Kit (Invitrogen #80–7064-88) and the Mouse TNF-alpha Uncoated ELISA Kit (Invitrogen # 88–7324-88), respectively.

For the isolation of total RNA from mouse liver, which had been infected with *A. baumannii* and treated with Tha-PA90 as mentioned earlier, the TaKaRa MiniBEST Universal RNA Extraction kit (TaKaRa #9767) was utilized according to the manufacturer’s instructions. The concentration and purity of the total RNA were determined using a spectrophotometer (DeNOVIX DS-11 FX). Subsequently, the isolated RNA was transcribed into cDNA using the Dyne cDNA Synthesis Kit (Dyne Bio # DYRT1120). Expression levels were assessed via qPCR analysis, employing a real-time PCR cycler (QIAGEN Roter-Gene Q) and TOPreal™ qPCR 2 × PreMIX (Enzynomics #RT500M). The primer sequences used for the reaction are presented in Table S1. Relative expression levels were determined using the ΔΔCt method, where ΔΔCt = ΔCt (experimental group)—ΔCt (control group). Cycle threshold (Ct) values were normalized to the Ct value of GAPDH, and changes relative to the mock were plotted. All experiments were conducted in triplicate.

### Statistical analysis

Data analysis was conducted using GraphPad Prism software version 9.3.0. A two-tailed Student’s t-test assessed the differences between the two groups, while survival experiments were analyzed using the log-rank (Mantel–Cox) test. All data are presented as the mean ± SD, and differences were considered to be statistically significant when *P* < 0.05.

## Results

### Thanatin fusion-improved antibacterial activity of endolysin PA90

**Fig. 1:**
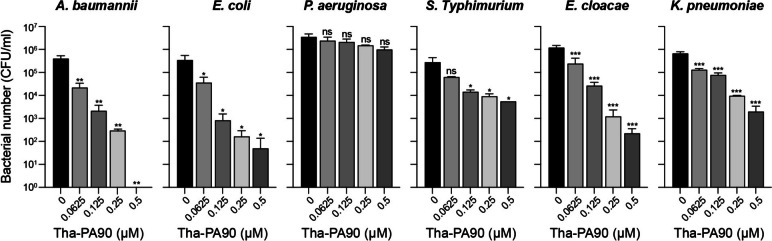
Antibacterial spectrum of Tha-PA90 against gram-negative pathogens. The antibacterial activity of Tha-PA90 was assessed via a CFU reduction assay using the following strains: *A. baumannii* ATCC 19606, *E. coli* MG1655, *P. aeruginosa* ATCC 13388, *S. Typhimurium* ATCC 14028 s, *E. cloacae* ATCC 13047, and *K. pn*e*umoniae* KCTC 2208. Exponentially grown bacterial cells were adjusted to 1 × 10^6^ CFU in 20 mM HEPES pH 7.4 and subsequently exposed to purified Tha-PA90 at concentrations of 0, 0.0625, 0.125, 0.25, and 0.5 μM at 37 ℃ for 2 h. The experiments were conducted independently at least thrice, and the data are presented as mean $\pm$ SD. Statistical significance was set at *p < 0.0449, **p < 0.0095, ***p < 0.0010, ns: not significant

In a previous study, we observed that the in vitro activity of PA90 was enhanced by adding the cell-penetrating peptide DS4.3 at the N-terminus [26]. However, this enhanced activity was not observed when applied to a mouse infection model (data not shown). In this study, we introduced a thanatin peptide (GSKKPVPIIYCNRRTGKCQRM) at the N-terminus of PA90 to improve bactericidal activity in vivo. Thanatin is an AMP known for its LPS-neutralizing activity and protective effects in a mouse sepsis model [23, 28]. To assess endolysin activity in vitro and in vivo, we overexpressed and purified the thanatin-fused PA90, called Tha-PA90, through affinity chromatography. Subsequently, we tested the purified Tha-PA90 for antibacterial activity using a CFU reduction assay. When Tha-PA90 was applied to bacterial cultures during the exponential growth phase, we observed a gradual reduction in the number of bacteria in proportion to the increased concentration of the recombinant endolysin (Fig. 1). Notably, differences were observed among the susceptibility levels of bacterial strains. Tha-PA90 led to less than a one-log decrease in *P. aeruginosa* or *S. Typhimurium*, corresponding to a 3.5- or 50-fold reduction, respectively. However, Tha-PA90 eliminated more than two log numbers of *K. pneumoniae* at a concentration of 0.5 μM. The same concentration of Tha-PA90 removed three log numbers of *E. cloacae* and *E. coli*. Most strikingly, *A. baumannii* was completely eradicated by treatment with 0.5 μM of Tha-PA90. Additionally, all tested MDR clinical isolates of *A. baumannii* [26], except for strain 3097, were eliminated using 0.5 μM of Tha-PA90 (Additional file 1: Fig. S1). Consequently, thanatin-fused PA90 exhibits robust antibacterial activity against most tested gram-negative pathogens, except for *P. aeruginosa* in vitro.

### Increased membrane permeability of Tha-PA90 leading to enhanced bactericidal activity

**Fig. 2:**
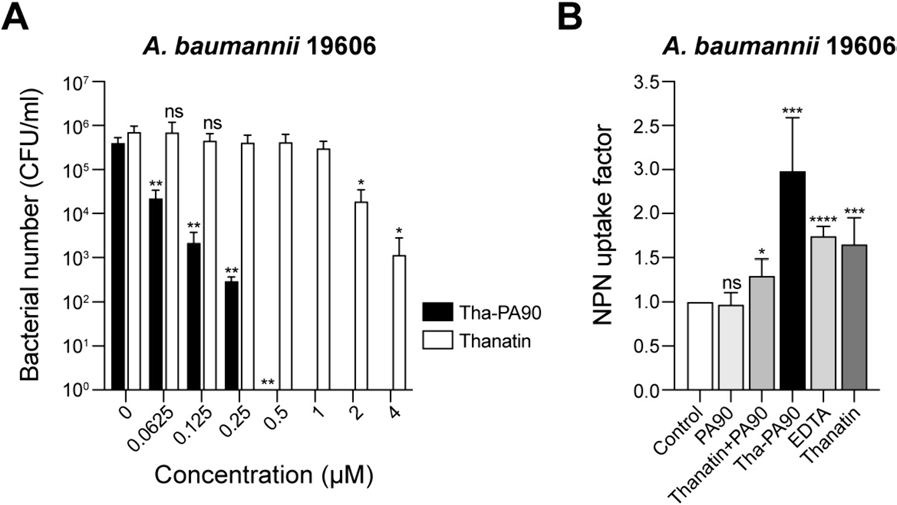
Enhanced antibacterial activity and permeability of Tha-PA90. **A** The antimicrobial activity of Thanatin or Tha-PA90 against *A. baumannii* ATCC 19606 was determined. Exponentially grown cells were exposed to the indicated concentrations of Tha-PA90 or Thanatin. Data are presented as mean $\pm$ SD (*n* = 3). Significance is indicated as *p < 0.0118, **p < 0.0095, n.s: not significant. **B** Membrane-penetrating activity of Tha-PA90 against *A. baumannii* ATCC 19606 was assessed through the NPN uptake assay. Bacterial cells were treated with none (control), PA90 (2 μM), a mixture of Thanatin and PA90 (Thanatin + PA90; 2 μM each), or Tha-PA90 (2 μM). EDTA was used as a membrane-disrupting chelator. The experiment was repeated three times. Significance is indicated as *p < 0.0124, ***p < 0.0005, ****p < 0.0001, ns: not significant

As thanatin possesses antibacterial activity, we compared the activity of Tha-PA90 and that of the thanatin peptide using *A. baumannii*. Remarkably, no bacteria survived in the presence of 0.5 μM of Tha-PA90. In contrast, up to three log numbers of bacteria could survive when exposed to 4 μM of thanatin peptide (Fig. 2A). This result underscores the superior activity of thanatin-fused PA90 compared with thanatin alone. To gain insight into the mode of action, we measured the level of NPN uptake that is indicative of membrane permeabilization, owing to the role of AMPs in disrupting bacterial membranes (Fig. 2B). NPN uptake was not observed in *A. baumannii* treated with buffer containing NPN only or with PA90 alone. When a mixture of thanatin peptide and PA90 was added, cell permeability increased 1.3-fold. EDTA or thanatin peptide also led to a 1.74-fold or 1.65-fold increase in NPN uptake, respectively. Strikingly, Tha-PA90 increased membrane permeability 2.48-fold, surpassing the effect of thanatin peptide alone. The fusion of thanatin to PA90 resulted in enhanced membrane permeability, ensuring improved accessibility of PA90 to its substrate, peptidoglycan, and consequently enhancing the antibacterial activity of the recombinant endolysin.

### Thanatin fusion to PA90 leading to epithelial cell penetration with no cytotoxic effect

**Fig. 3:**
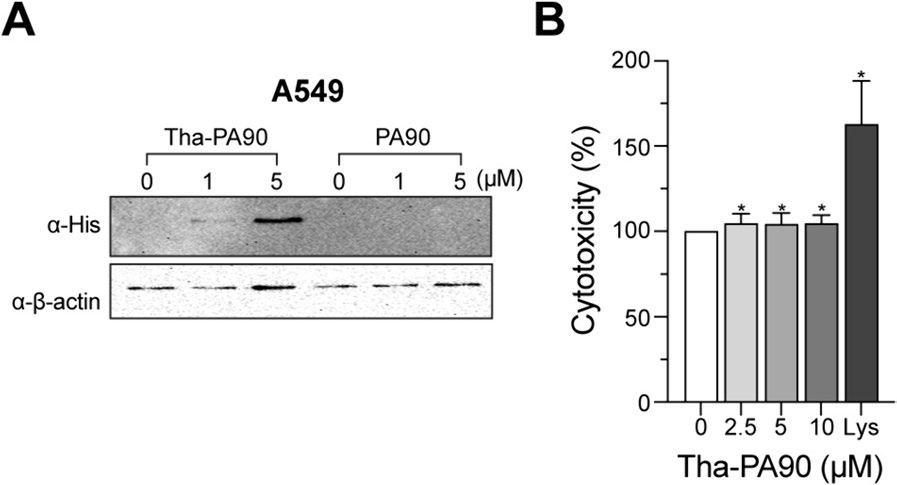
Internalization and cytotoxicity of Tha-PA90 in A549 cells. **A** Western blot analysis of internalized endolysins. A549 cells were incubated with 0, 1, and 5 μM of Tha-PA90 or PA90. After a 30 min incubation, cells were washed with DPBS to remove external proteins and harvested in 0.05% Triton X-100 solution. Western blotting was performed using an anti-His antibody, with beta-actin as a control. **B** A549 cells were exposed to 2.5, 5, or 10 μM of Tha-PA90 for 24 h. The cell viability was assessed using the lactate dehydrogenase (LDH) assay. The control group was treated with cell lysis buffer. The experiment was repeated three times. Statistical significance was set at *p < 0.05

Subsequently, we assessed the impact of thanatin fusion on the ability of PA90 to penetrate epithelial cells owing to thanatin’s cell-penetrating peptide properties. Following a 30 min incubation at 37 °C, Tha-PA90 was detected in the intracellular fraction of A549 cells. In contrast, regardless of its concentration, PA90 was not detected inside the cells. Notably, higher concentrations of Tha-PA90 led to more protein being detected within the cells (Fig. 3A). Furthermore, we conducted a lactate dehydrogenase (LDH) assay to evaluate cytotoxicity. The results demonstrated that A549 cells exhibited no cytotoxicity when treated with Tha-PA90 at concentrations of up to 10 μM (Fig. 3B). A549 cells showed minimal impact in the presence of Tha-PA90 for 24 h while adding mammalian cell lysis buffer led to a 1.63-fold increase in LDH release. These findings suggest that Tha-PA90 can potentially target and affect infected bacteria without causing damage to mammalian cells.

### Prolonged survival of mouse with *A. baumannii* infection on administration of Tha-PA90

**Fig. 4:**
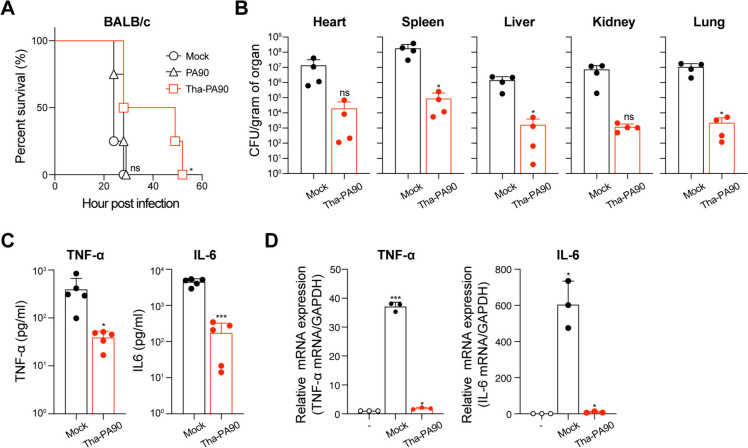
In vivo efficacy of Tha-PA90 in a mouse model of *A. baumannii* systemic infection. **A** BALB/c mice, treated with CPM, were intraperitoneally injected with 1 × 10.^8^ CFU of *A. baumannii* ATCC 19606. One hour post infection, the animals received an intraperitoneal injection of mock (1 × PBS), PA90, or Tha-PA90 (600 μg/mice). Survival rates were monitored for 52-h. **B** After 10 h post-infection, mice were euthanized, and organs were isolated to enumerate bacterial load by plating serial dilutions of homogenized organs. **C** Serum levels of IL-6 or TNF-α in mock or Tha-PA90-treated mice were analyzed with ELISA 10-h post-injection. Significance is indicated as ***p < 0.0006, *p < 0.0468. **D** Expression levels of IL-6 or TNF- α from liver tissues were evaluated by measuring mRNA relative to GAPDH qPCR. All experiments were performed in triplicate. Significance is indicated as ***p < 0.0006, *p < 0.0463

We then evaluated the potential of Tha-PA90 as an alternative to antibiotics using a mouse sepsis model of *A. baumannii* infection. The mouse model was established by intraperitoneal injection of *A. baumannii* 19606 following cyclophosphamide-induced neutropenia [29]. Endolysins were administered via the intraperitoneal route 1-h after infection. Notably, all infected mice treated with 1 × PBS (mock) or PA90 succumbed within 30-h. In contrast, the lifespan was extended to up to 52-h with Tha-PA90 treatment (Fig. 4A). Furthermore, bacterial burdens in five major organs (heart, spleen, liver, kidney, and lung) were significantly reduced, up to 4 log CFU/gram of organ, 10-h after the administration of Tha-PA90 (Fig. 4B). Given that thanatin blocks the LPS-stimulated TLR4 signaling pathway, resulting in the inhibition of proinflammatory cytokine induction [28], we investigated the levels of such cytokines upon Tha-PA90 treatment in mice with *A. baumannii* sepsis. Tha-PA90 treatment led to a substantial decrease, up to tenfold for TNF-α and 26-fold for IL-6, compared with administration of 1 × PBS, as observed in the serum from infected mice (Fig. 4C). Similarly, the expressions of TNF-α and IL-6 were reduced by 20-fold and 43-fold, respectively, in the liver of mice treated with Tha-PA90 relative to non-treated mice with *A. baumannii* infection (Fig. 4D). Notably, the enhanced antibacterial activity of Tha-PA90 was also observed in mouse infection models involving other gram-negative pathogens or multiple species. In an intraperitoneal injection model using *E. coli* MG1655, more than a 70% increase in survival was observed in the group of mice treated with Tha-PA90 (Additional file 1: Fig. S2). In a cecal ligation puncture (CLP) mouse model for polymicrobial sepsis, the lifespan of mice was extended to 75-h following Tha-PA90 administration. In contrast, mock-treated mice succumbed within 29-h, and those treated with PA90 lived up to 50-h (Additional file 1: Fig. S3). Tha-PA90 effectively controls gram-negative pathogens, resulting in improved survival without inducing excessive production of proinflammatory cytokines.

## Discussion

AMP-fused endolysins exhibit superior efficacy compared to native endolysins when targeting gram-negative pathogens with compromised outer membranes owing to AMP-induced outer membrane disruption. This disruption enhances endolysin access to the underlying peptidoglycan [26, 30–32]. In a previous study, we engineered the newly isolated endolysin PA90 by incorporating the cell-penetrating peptide DS4.3 to augment its enzymatic activity, resulting in DS-PA90 [26]. In vitro experiments demonstrated that *A. baumannii* were all killed by exposure to 0.25 μM of DS-PA90, and insect larvae infected by *A. baumannii* could survive when treated with 10 μM of DS-PA90. However, no antibacterial activity was observed in a mouse model of *A. baumannii* infection. This failure might be attributed to the sudden release of lipopolysaccharide (LPS) following post-treatment bacterial lysis, leading to cytokine storms and eventual mortality. To address this issue, we modified the fusion peptide from DS4.3 to thanatin to neutralize and aggregate LPS.

In CFU reduction assays, Tha-PA90 effectively eradicated *A. baumannii* and *E. coli*, with a dose-dependent, moderate effect observed against *S. typhimurium*, *E. cloacae*, and *K. pneumoniae*. Notably, *P. aeruginosa* did not exhibit clear susceptibility to Tha-PA90 under our experimental conditions. This differential response of Tha-PA90 against various bacterial strains may be attributed to disparities in their membrane and cell wall compositions. Furthermore, we observed superior efficacy of Tha-PA90 compared to the antibacterial activity of thanatin peptide alone in vitro, particularly against *A. baumannii*, as demonstrated in CFU reduction assays. This enhanced activity is likely attributable to the increased cell permeability of Tha-PA90, as evidenced by NPN uptake assays, which showed significantly higher permeability compared to PA90, thanatin, and a mixture of thanatin and PA90. These results suggest that an endolysin can rapidly access its substrate following bacterial membrane disruption by fused antimicrobial peptides, resulting in rapid enzymatic action. We assessed the potential of Tha-PA90 as a therapeutic agent for bacterial infections in vivo, utilizing a mouse infection model. Initially, mild neutropenia was induced by administering CPM to ensure infection by *A. baumannii*, an opportunistic pathogen [29, 33]. All mice infected with *A. baumannii* succumbed within 30-h, and no survival benefit was observed among those treated with PA90. Surprisingly, when treated with Tha-PA90, half the infected mice survived for up to 52-h, demonstrating the in vivo antibacterial efficacy of Tha-PA90.

Furthermore, we observed a fourfold reduction in bacterial counts in major organs of infected mice. Additionally, Tha-PA90 treatment led to a remarkable 26-fold decrease in proinflammatory cytokine levels in the serum, with a corresponding 45-fold reduction in cytokine expression in the infected mice. This substantial reduction in proinflammatory cytokines suggests that pathogen-associated molecular patterns (PAMPs), including LPSs, were not excessively released upon bacterial lysis due to the action of Tha-PA90. In an acute *E. coli* infection mouse model, Tha-PA90 provided stronger protection, resulting in a 70% increase in survival rates. Furthermore, Tha-PA90 extended the lifespan of mice by up to 2.5-fold in a multi-species infection model induced by the CLP procedure. The varying susceptibility observed may be contingent upon the distinct pathogenic mechanisms and immune evasion strategies employed by each bacterium within the mammalian host. Our in vivo data support the potential applicability of Tha-PA90 as an alternative to antibiotics, with a reduced risk of inducing cytokine storms, irrespective of bacterial species.

## Conclusions

In this study, we demonstrated the antibacterial efficacy of an engineered endolysin fused with an LPS-disrupting peptide both in vitro and in vivo. However, several considerations must be addressed before considering engineered endolysins as alternative antibiotics in clinical settings. First, efforts should be directed toward improving the in vivo efficacy, as it is not yet as effective as conventional antibiotics. Notably, the low salt tolerance of endolysin is a challenge that needs to be addressed, as it renders the endolysin ineffective in saline solutions [26, 30, 34]. Potential solutions may involve additional protein engineering, such as fusion with different types of peptides or modification of amino acid sequences [35, 36].

Moreover, exploring the synergy between engineered endolysins and clinically available antibiotics is essential to enhancing therapeutic efficacy and mitigating the development of resistance against either antibiotics or endolysins. While there are no reports of obvious resistance to endolysins to date [37, 38], resistance mechanisms should be investigated both in vitro and in vivo. Finally, comprehensive assessments of in vivo toxicity and immune responses are imperative when introducing engineered endolysins via various routes, including intravenous injection, to ensure clinical safety.

### Supplementary Information

**Supplementary Material 1.**

## Data Availability

The data sets used during the current study are accessible from the corresponding author upon reasonable request.

## Abbreviations

AMP: Antimicrobial peptide
Tha: Thanatin
TNF-α: Tumor necrosis factor-alpha
IL-6: Interleukin-6
CFU: Colony forming unit
LPS: Lipopolysaccharide
MDR: Multi-drug-resistant
XDR: Extensively drug-resistant
PDR: Pandrug-resistant
IPTG: Isopropyl-B-D-thiogalactopyranoside
RT: Room temperature
LDH: Lactate dehydrogenase
ELISA: Enzyme Linked Immunosorbent assay
CPM: Cyclophosphamide
BHI: Brain heart infusion
qPCR: Quantitative polymerase chain reaction
NPN: 1-N-phenylnaphthylamine
EDTA: Ethylenediaminetetraacetic acid
DMEM: Dulbecco’s modified Eagle’s medium
FBS: Fetal bovine serum
