# Supplementary material for: Antimicrobial peptide thanatin fused endolysin PA90 (Tha-PA90) for the control of Acinetobacter baumannii infection in mouse model
Source: J Biomed Sci. 2024 Apr 15;31:36. doi: 10.1186/s12929-024-01027-4 (PMC11020296; doi:10.1186/s12929-024-01027-4)
Supplement: Supplementary file 1 — Supplementary Material 1. [file 12929_2024_1027_MOESM1_ESM.docx]

Table S1. Primers used in the study.

| Name | Sequences |
| --- | --- |
| Primers for the construction of pAS036 (pET21a::Thanatin::PA90) | |
| Tha iF | 5’-GTAATCGTCGCACAGGGAAGTGCCAGCGGATGATGGGTACTGTACTCAAACGTGGC-3’ |
| Tha iR | 5’-AATAAATAATAGGAACCGGTTTCTTGCTACCCATATGTATATCTCCTTCTTAAAGTTAAACAAAATTATTTC-3’ |
| Primers for qPCR | |
| TNF F | 5’-CCTGTAGCCCACGTCGTAG-3’ |
| TNF R | 5’- GGGAGTAGACAAGGTACAACCC-3’ |
| IL-6 F | 5’- AACGATGATGCACTTGCAGA-3’ |
| IL-6 R | 5’- GAGCATTGGAAATTGGGGTA-3’ |
| GAPDH F | 5’- CCCACTAACATCAAATGGGG-3’ |
| GAPDH R | 5’- CCTTCCACAATGCCAAAGTT-3’ |

* Nucleotide sequences for thanatin peptide are underlined.


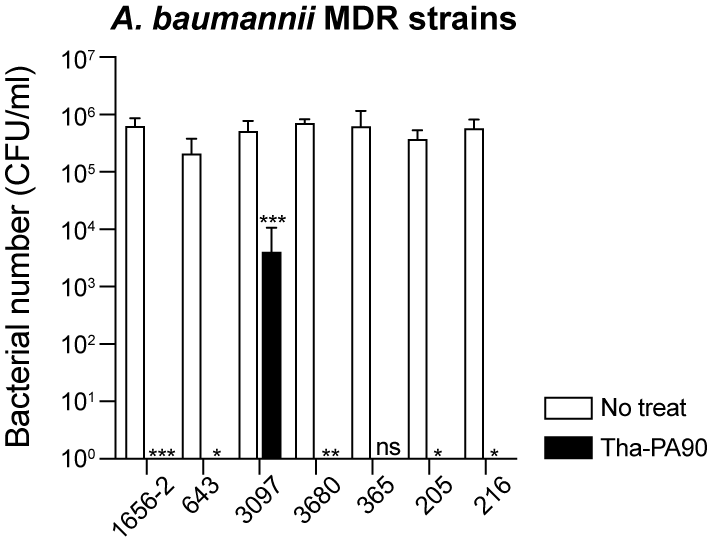


**Fig. S1. Antibacterial activity of Tha-PA90 against *A. baumannii* MDR strains.** MDR strains of *A. baumannii* [1] were used for CFU reduction assay to assess the endolysin activity of Tha-PA90. Exponentially grown cells were adjusted to 1 × 10^6^ CFU in 20 mM HEPES pH 7.4 and treated with 0, 0.125, 0.25, and 0.5 μM of purified Tha-PA90 at 37 ℃ for 2 h. The experiments were repeated at least three times and data are presented as mean $\pm$ SD. Significance is shown as *p < 0.0172, **p< 0.0085, ***p < 0.0007, ns : not significant.


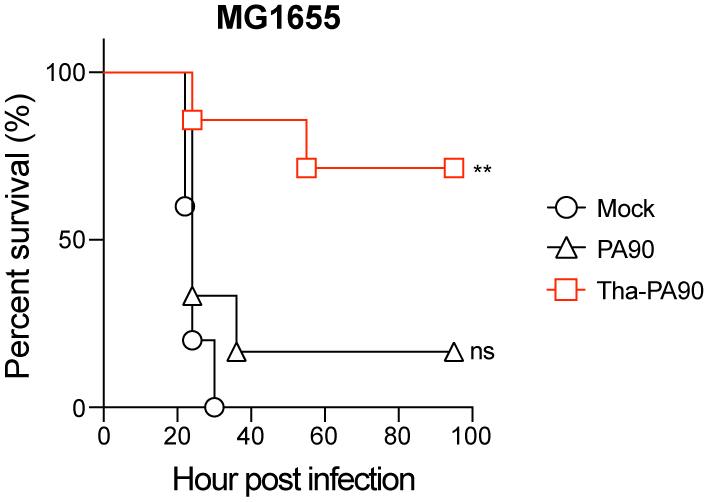


**Fig. S2. Antibacterial activity of Tha-PA90 in a mouse model of *E. coli* systemic infection.** BALB/c mice were injected intraperitoneally with 2 × 10^8^ CFU of MG1655. One hour post infection, animals received an intraperitoneal injection of mock (1 x PBS), PA90 or Tha-PA90 (600 μg/mice). The survival rates were monitored for 95 h. Significance is indicated as **p = 0.0028, ns : not significant.


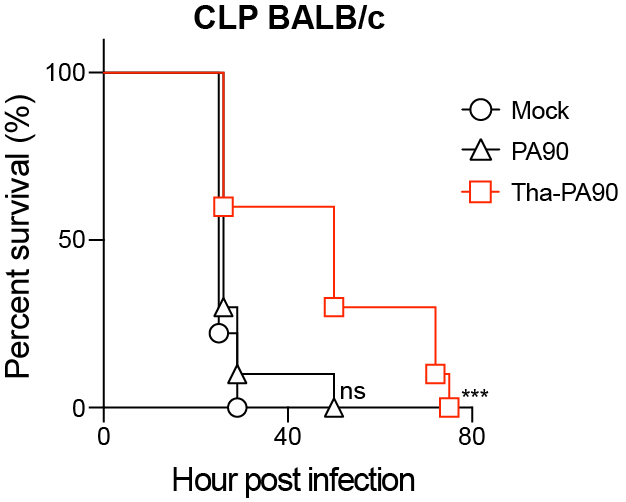


**Fig. S3. In vivo efficacy of Tha-PA90 in mouse model for cecal ligation puncture (CLP)-induced polymicrobial sepsis.** The CLP procedure was performed as described [2]. After administration of isoflurane anesthesia, an incision was made in the abdomen, where the cecum was externalized and ligated. A puncture was subsequently introduced with a 23-Gauge needle; metallic clips were used for closing the incision. One hour after CLP, mice received an intraperitoneal injection of mock (1 x PBS), PA90 or Tha-PA90 (600 μg/mice). The survival rates were monitored for 75 h. Significance is indicated as ***p = 0.0003, ns : not significant.

References

1. J L, J J, H M, M S. Eradication of drug-resistant Acinetobacter baumannii by cell-penetrating peptide fused endolysin. J microbiol. 2022;60:859-866

2. Dejager L, Pinheiro I, Dejonckheere E, Libert C. Cecal ligation and puncture: the gold standard model for polymicrobial sepsis? Trends Microbiol. 2011;19:198–208.
